# A Rare Occurrence of Tularemia in New Jersey

**DOI:** 10.7759/cureus.18329

**Published:** 2021-09-27

**Authors:** Dhairya Gor, Kyle Wiseman, Christopher Lesniak, Edward Liu

**Affiliations:** 1 Internal Medicine, Jersey Shore University Medical Center, Neptune, USA

**Keywords:** rare infections, rabbits, new jersey, francisella tularensis, tularemia

## Abstract

Tularemia has been well described clinically in the United States since the early 1900s. Worldwide, the infection has manifested in a variety of forms through various vectors with geography and local prevalence often considered in the diagnostic work-up. We present a case of a 57-year-old patient who lived in an area with low tularemia infection rates and presented with fever and a tender, swollen elbow. Though initially diagnosed with cellulitis, she was eventually found to have tularemia after further interviewing and questioning, followed by successful treatment with doxycycline. A thorough history including exposures and daily activities should always be considered in an effort to rule out rare infections, even in areas of low disease prevalence.

## Introduction

Tularemia is an infectious disease that originates from the aerobic, gram-negative facultative intracellular bacterium *Francisella tularensis* [1]. While first reported as the cause of tularemia in Ohio in 1914, previous accounts of clinical presentations resembling it from as early as 1653 have been reported [2]. The overall incidence of tularemia has decreased in the United States and is currently 0.07/100,000, with only one case reported in New Jersey in 2018 [3]. Presentation is often non-specific, with fevers, chills, myalgias, headache, and lymphadenopathy [1,4,5]; however, delay in diagnosis due to its rarity in non-endemic areas can lead to systemic complications. In this report, we present the rare case of a 57-year-old female living in New Jersey, diagnosed with tularemia.

## Case presentation

A 57-year-old woman initially presented to an urgent care center with a two-week history of fever and a tender swollen lesion near her right elbow that worsened over four days. She was diagnosed with cellulitis and treated with cephalexin. After two more days of persistent fever and worsening arm erythema, she presented to the emergency department. She denied any trauma, insect bites, or systemic symptoms including headaches, nausea, vomiting, diarrhea, dysuria, shortness of breath, or cough. Computed tomography (CT) imaging of the right upper extremity showed multiple enlarged lymph nodes, with the largest measuring 3.0 x 2.7 x 3.9 cm in the right axilla with a 12 mm spherical structure in the subcutaneous fat with surrounding erythema. Again she was treated for cellulitis with IV clindamycin and discharged home on amoxicillin-clavulanic acid. One week later she returned to the emergency department with persistent fever. She endorsed worsening right axillary pain but denied weight loss, night sweats, or bone pain. She also reported that she owned a dog and would often feed stray cats at her home. 

**Figure 1:**
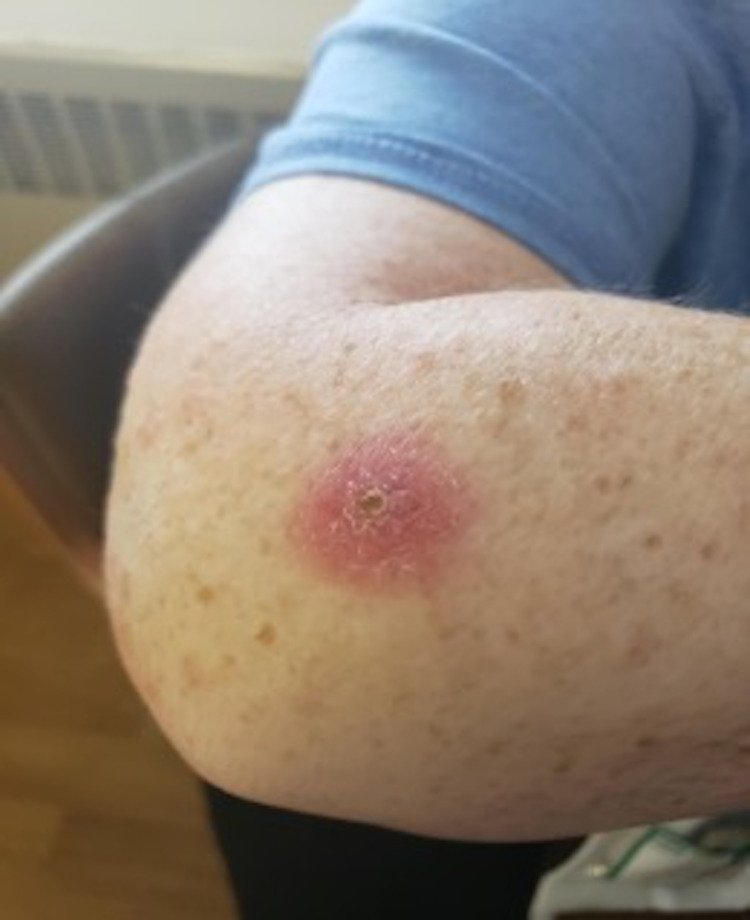
Indurated, erythematous lesion located near the patient's right elbow.

In the emergency department, blood pressure was 100/79 mmHg, heart rate 112/minute, oxygen saturation 99% on room air, and temperature 99.4^o^ Fahrenheit orally. On physical examination, the patient had a tender, nodular lesion in the right axilla about 2 cm in diameter with surrounding erythema. Also noted was a lesion lateral to the right elbow with induration and a scab in the center with surrounding erythema (Figure 1). Labs were significant for a leukocyte count of 15.9 x 10^3^/μL, and absolute neutrophil count of 11.5 x 10^3^/μL with normal hemoglobin and platelets. Serum electrolytes, liver function, kidney function, and lactate were within normal limits. Ultrasound imaging of the arm revealed a 1.38 x 1.4 x 1.37 cm soft tissue structure with increased echogenicity centrally and a hypoechoic rim with no associated hypervascularity. CT scan of the chest/abdomen/pelvis showed multiple lymph nodes in the right axilla, with the largest measuring 5.1 x 3.2 x 4.4 cm and mild hepatomegaly. The spleen was normal in size. The patient was started on vancomycin initially and then doxycycline was added. Vancomycin was discontinued as blood cultures came back negative. Her fever improved on doxycycline within 24 hours.

Further workup showed that *Ehrlichia* IgG was elevated, but IgM was negative. Lyme polymerase chain reaction (PCR), *Bartonella* PCR, *Yersinia* culture, and *Rickettsia* antibodies were all negative. A biopsy of the axillary lesion was performed with fungal, acid-fast bacteria and bacterial culture specimens sent. The gram stain was negative for any bacteria or white blood cells. Flow cytometric analysis showed no immunophenotypic evidence of non-Hodgkin lymphoma. The histopathology report showed necrotizing granulomas and focal nodular paracortical hyperplasia. The patient was discharged on doxycycline for possible tick borne disease. The patient was followed as an outpatient and the diagnosis was confirmed with positive *Francisella tularensis* antibodies, IgM 12 u/mL and IgG 70 u/mL with resolution of symptoms using twice-daily 100 mg doxycycline for 21 days. When the tularemia diagnosis was discussed with the patient, she revealed that her lawn was frequently visited by rabbits and she did yard work regularly in the area.

## Discussion

Tularemia is an infectious disease that originates from the bacterium *Francisella tularensis* [1]. The aerobic, gram-negative facultative intracellular organism was first reported as the cause of tularemia in Ohio in 1914, with previous accounts of clinical presentations resembling it from as early as 1653 [2]. Initially isolated in 1911, cases of tularemia have been reported all over the world. The highest number of cases in the United States are currently in Arkansas, and most cases, in general, seem to appear in the Southern, Central, and Southwestern states [2]. The overall incidence of tularemia has decreased in the United States and is currently 0.07/100,000 as of 2018, with only one case being reported in New Jersey in 2018 [3]. 

Four biotypes of tularemia exist, including tularensis, holarctica, mediasiatica, and novicida. Up to 70% of reported cases are caused by the North American strain of the tularensis subspecies, also known as Type A [2]. Type A is the more virulent and deadly form in humans, [6] and its primary reservoirs are cottontail rabbits (sylvilagus species) and multiple species of ticks [2]. However, other small rodents and even cats have been implicated as potential additional sources in the United States [4,7]. The holarctica subspecies (also known as Type B) reside in aquatic rodents such as beavers and are the most common cause of tularemia in Europe, though it is much less virulent [8]. Human infection has been rare in novicida and yet to be reported in mediasiatica [7]. 

Tularemia can be transmitted to humans in multiple ways, including handling infected animals, consuming contaminated food/water, tick bites, fleas, contact with the aquatic environment, and inhalation via aerosols [4,7]. Mosquitoes have also been recently identified as additional vectors for *Francisella tularensis holarctica*, particularly in Northern Europe during the warm season [9]. Human-to-human transmission is sporadic, and the most common mode of transmission is through human skin contact [1,7]. Tularemia has shown the ability to be transmitted in respiratory droplets and the pathogen is highly contagious and can be deadly; thus, it is classified as a Category A bioterrorism agent [2]. The high transmissibility of tularemia has been further demonstrated by the notable Martha's Vineyard outbreak in Massachusetts in 2000 [10].

Six clinical forms of tularemia exist, including an ulcerative-glandular for,, which is the most common form [5,7,8,11]. The ulcerative-glandular form generally has associated lymphadenopathy, whereas isolated lymphadenopathy that lacks an ulcer has been termed the “glandular” form. Other forms include oropharyngeal, oculoglandular, and severe typhoidal tularemia [4]. The sixth and final clinical form is respiratory/pneumonic tularemia transmitted via respiratory droplets [5,7]. Clinically, the pneumonic form of tularemia has a more insidious presentation of chronic coughs, fevers, weight loss, and mediastinal lymphadenopathy resembling tuberculosis and sarcoidosis [4]. Notably, the pneumonic and typhoidal tularemia are bloodstream infections and have had US death rates upward of 60% in the Type A.1 strain, as reported in a 1992 study [7]. 

Tularemia generally presents after a roughly 3-5 day incubation period with nonspecific symptoms of fever, chills, myalgia, arthralgia, and headache [1,4,5]. Our patient initially reported fever and a tender erythematous lesion on the right arm. She later reported exposure to rabbits in her yard. The clinical picture of fever with lymphadenopathy and exposure to a vector or animal can lead to suspicion of tularemia, along with other differential diagnoses including Q fever, plague, and psittacosis [7]. The diagnosis of tularemia is through serological antibody tests and rarely through bacterial culture, as done in our patient. Antibodies often develop 2-3 weeks after the initial infection and are often used for detecting *Francisella tularensis* [1,7]; however, ELISA and microagglutination tests are the most commonly used, although PCR can be used as well [1]. Generally, a four-fold increase in anti-*Francisella* serum antibody within 2-4 weeks can be used to confirm infection [7].

Complications from tularemia can lead to a multitude of ongoing tissue infections, most notably lymphadenopathy leading to lymph node suppuration, prolonging the diseased state [4]. Prognosis is often worse in immunocompromised patients or those who did not receive immediate treatment [4]. Death rates in the United States from tularemia generally vary by the subspecies genotype, with more recent studies showing a Type A1b reporting rate of 24% in 2009 [4].

The World Health Organization recommends aminoglycosides as treatment for tularemia, such as gentamicin 5 mg/kg daily every 12 hours for 10 days or streptomycin 2 g intramuscularly every 12 hours for 10 days [8]. In milder cases, doxycycline 200 mg daily for 15 days is often used [8]. In addition, ciprofloxacin is effective as well at a dosing of 800-1000 mg daily divided into a morning and nighttime dose over 14 days [8]. Our patient had mild symptoms and was treated with doxycycline with resolution of symptoms over the next two weeks. 

This case highlights a rare case of tularemia showing the importance of including it in the differential diagnosis even in nonendemic areas like New Jersey. The diagnosis can be challenging with a nonspecific presentation and should be considered in patients with fever, regional lymphadenopathy, and a history of contact with a small rodent, tick, or mosquito. It is essential to start the antibiotics when first suspected, as the serology test will take time to result. Proper antibiotic administration leads to a favorable prognosis as it can be fatal if not treated promptly.

## Conclusions

Though a rare diagnosis in many areas of the United States, tularemia can still show up in nonendemic areas. Some forms are particularly of interest given the high associated morbidity. Additionally, tularemia has been shown to transmit via multiple vectors beyond the classical transmission patterns. Thus, when a patient presents with persistent fever despite empiric antibiotic management, consideration of environmental exposures along with an appropriate physical examination should be re-visited to broaden the differential diagnosis.
